# Ethnicity Differences in Sleep Changes Among Prehypertensive Adults Using a Smartphone Meditation App: Dose-Response Trial

**DOI:** 10.2196/20501

**Published:** 2020-10-06

**Authors:** John C Sieverdes, Frank A Treiber, Christopher E Kline, Martina Mueller, Brenda Brunner-Jackson, Luke Sox, Mercedes Cain, Maria Swem, Vanessa Diaz, Jessica Chandler

**Affiliations:** 1 College of Charleston Health and Human Performance Charleston, SC United States; 2 College of Nursing Medical University of South Carolina Charleston, SC United States; 3 College of Medicine Medical University of South Carolina Charleston, SC United States; 4 Department of Health & Physical Activity University of Pittsburgh Pittsburgh, PA United States

**Keywords:** meditation, sleep, mobile phone, prehypertension, ethnicity

## Abstract

**Background:**

African Americans (AAs) experience greater sleep quality problems than non-Hispanic Whites (NHWs). Meditation may aid in addressing this disparity, although the dosage levels needed to achieve such benefits have not been adequately studied. Smartphone apps present a novel modality for delivering, monitoring, and measuring adherence to meditation protocols.

**Objective:**

This 6-month dose-response feasibility trial investigated the effects of a breathing awareness meditation (BAM) app, Tension Tamer, on the secondary outcomes of self-reported and actigraphy measures of sleep quality and the modulating effects of ethnicity of AAs and NHWs.

**Methods:**

A total of 64 prehypertensive adults (systolic blood pressure <139 mm Hg; 31 AAs and 33 NHWs) were randomized into 3 different Tension Tamer dosage conditions (5,10, or 15 min twice daily). Sleep quality was assessed at baseline and at 1, 3, and 6 months using the Pittsburgh Sleep Quality Index (PSQI) and 1-week bouts of continuous wrist actigraphy monitoring. The study was conducted between August 2014 and October 2016 (IRB #Pro00020894).

**Results:**

At baseline, PSQI and actigraphy data indicated that AAs had shorter sleep duration, greater sleep disturbance, poorer efficiency, and worse quality of sleep (range *P*=.03 to *P*<.001). Longitudinal generalized linear mixed modeling revealed a dose effect modulated by ethnicity (*P*=.01). Multimethod assessment showed a consistent pattern of NHWs exhibiting the most favorable responses to the 5-min dose; they reported greater improvements in sleep efficiency and quality as well as the PSQI global value than with the 10-min and 15-min doses (range *P*=.04 to *P*<.001). Actigraphy findings revealed a consistent, but not statistically significant, pattern in the 5-min group, showing lower fragmentation, longer sleep duration, and higher efficiency than the other 2 dosage conditions. Among AAs, actigraphy indicated lower sleep fragmentation with the 5-min dose compared with the 10-min and 15-min doses (*P*=.03 and *P*<.001, respectively). The 10-min dose showed longer sleep duration than the 5-min and 15-min doses (*P*=.02 and *P*<.001, respectively). The 5-min dose also exhibited significantly longer average sleep than the 15-min dose (*P*=.03).

**Conclusions:**

These findings indicate the need for further study of the potential modulating influence of ethnicity on the impact of BAM on sleep indices and user-centered exploration to ascertain the potential merits of refining the Tension Tamer app with attention to cultural tailoring among AAs and NHWs with pre-existing sleep complaints.

## Introduction

### Background

Obtaining an adequate amount and quality of sleep is essential for optimal health. Chronically insufficient sleep negatively impacts daytime function and has been linked to an increased risk of hypertension, stroke, cardiovascular disease, obesity, stress, and poor mood [1-6]. One of the most common contributors to poor sleep is chronic stress [7-11], which can disrupt sleep by increasing rumination, worries, intrusive thoughts, and heightened muscular tension [12,13]. Mind-body strategies addressing stress may not only aid in the improvement of sleep quality but also reduce exacerbation of other chronic conditions such as hypertension, cardiovascular disease, diabetes, and obesity [14-16].

Mitigating stress through positive cognitive behavioral coping strategies is a common treatment approach to improve sleep quality [11]. Meditation is one tactic that can be incorporated into a comprehensive treatment plan among those with chronic stress experiencing trouble sleeping [8,10]. A variety of meditation approaches already exist, including transcendental meditation (TM) and breathing awareness meditation (BAM). There is evidence to show improvements in anxiety, stress [17-19], and blood pressure (BP) through the use of these types of meditation techniques [20-23]. These practices focus on relaxation, self-acceptance, and staying in the present moment through slow, diaphragmatic breathing.

Although the benefits of meditation on stress are positive, sleep outcomes have been mixed. Neuendorf et al [11] reviewed 112 mind-body–based randomized controlled trials (RCTs) on sleep quality outcomes. Of the 23 meditation RCTs, only 39% reported beneficial effects on sleep quality. A 2016 meta-analysis of 6 RCTs using mindfulness meditation incorporating BAM reported statistically significant but mild improvements in self-reported total wake time, sleep onset latency, sleep efficiency, and sleep quality [24]. Another meta-analysis of 47 RCTs found minimal evidence of meditation studies improving positive mood, attention, eating habits, or sleep [17]. Collectively, the findings are somewhat mixed, with mild-to-moderate effects of meditation interventions on sleep quality. Potential reasons for the variability in outcomes included heterogeneity of participants, with some studies using individuals identified as having sleep disorders, some studies using patients with mental health disorders associated with sleep disturbances (eg, anxiety, depression, posttraumatic stress disorder [PTSD]), and others assessing sleep outcomes among individuals not recruited for sleep issues. Many of these studies were conducted for a short term (eg, 8-12 weeks), often relied upon self-report rather than multimethod assessment (eg, inclusion of polysomnography and actigraphy), did not objectively monitor adherence to meditation protocols, and failed to report study details such as the level of expertise of the trainers and the degree of blinding of the evaluators. [11,13,24]. Furthermore, none of the RCTs reviewed were designed to evaluate dose-response effects on sleep disturbance indices.

Large-scale dissemination of meditation programs using traditional modes of training may be challenging. Individual or group-based in-person training and monitoring methodologies have typically been used [11,17]; however, these approaches often result in challenges of travel, child care, and other logistical barriers that may reduce participation and retention rates. Finally, not having an objective indicator of adherence to meditation sessions outside of the in-person sessions has been noted as a potential contributor to the variability in outcomes across studies [11,13,24].

Technology-delivered meditation programs are an innovative option to overcome several of these barriers. Unfortunately, few programs have been rigorously evaluated. In particular, meditation programs delivered by smartphone apps are widely available [25] and can be used for reducing stress and for other clinical uses such as corresponding reductions in BP and improved sleep. However, the effects of these meditation apps on sleep indices have received sparse empirical evaluation [26].

Research has also consistently shown greater self-reported sleep quality problems (eg, longer latency of onset, shorter duration, greater disruption during sleep) among African Americans (AAs) than non-Hispanic Whites (NHWs) [3,27-31]. These findings have typically been supported in studies that included both objective (eg, wrist actigraphy, polysomnography) and self-reported measures [32-35]. The Tension Tamer study’s use of self-reported and actigraphy-based sleep assessment will aid in determining whether ethnic differences in sleep quality are observed via both self-reported and objective measurements. The available data enable a preliminary exploration of whether ethnicity potentially modulates prehypertensive adult responsivity to varying doses of the Tension Tamer app upon sleep.

Further studies are warranted to determine if meditation techniques, such as BAM, have a beneficial dose-response influence upon indices of sleep based on self-reported and objective verification via accelerometry. A recently completed 6-month dose-response study using Tension Tamer, a BAM smartphone app, provides this opportunity. The primary goal of the feasibility trial was to determine the potential dose-response impact on resting BP among prehypertensive adults [23]. We examined the reduction of resting systolic BP (SBP) compared with baseline levels for the 3 twice-daily doses (5, 10, and 15 min) of using the app at each evaluation point (1, 3, and 6 months). All dosage conditions had statistically significant reductions in BP, with 5-min and 10-min conditions showing average reductions in SBP (−5 to −8 mm Hg) comparable with other BAM and TM trials with prehypertensive and hypertensive samples [23,36-41]. The 15-min dose condition showed larger average SBP reductions compared with the 5-min and 10-min dose conditions, ranging from −10.9 mm Hg to −12.4 mm Hg across the 6-month trial. Secondary analyses are now directed at evaluating the potential impact of varying BAM doses on known modulators of BP control, such as sleep quality [42-44].

### Objectives

This paper reports on the exploratory analysis of 3 different doses of BAM on sleep quality changes in adult NHWs and AAs with prehypertension. We hypothesized that increased doses of BAM would impart greater improvements in self-reported and actigraphy-derived measures of sleep quality. We also hypothesized that AAs would exhibit poorer baseline levels of sleep quality compared with NHWs.

## Methods

### Participants

A total of 287 prehypertensive adults were recruited using paper advertising and word-of-mouth referrals at an academic health center in southeastern United States. Study candidates were contacted by phone to initiate eligibility screening and were scheduled for the first of 3 clinic BP evaluations. Inclusion criteria included participants aged between 18 and 90 years; being NHWs or AAs; and having systolic prehypertension based on the Seventh Report of the National Committee on Prevention, Detection, Evaluation, and Treatment of High Blood Pressure (120 mm Hg-139 mm Hg) on 3 consecutive visits, each at least 2 days apart. Exclusion criteria included unwillingness to accept randomization into 1 of 3 dosage groups, current medication regimens that affected BP, inability to open and navigate the Tension Tamer smartphone app after a hands-on training, or inability of participants to position their fingers over their smartphone camera because of tremors. Additional exclusion criteria included being currently pregnant, lactating, or having intention to become pregnant during the trial, participating in another research study of any kind, inability to read or speak English, and not having smartphone signal at the participant’s place of residence. The study was conducted between August, 2014 and October, 2016.

### Description of Tension Tamer App

**Figure 1:**
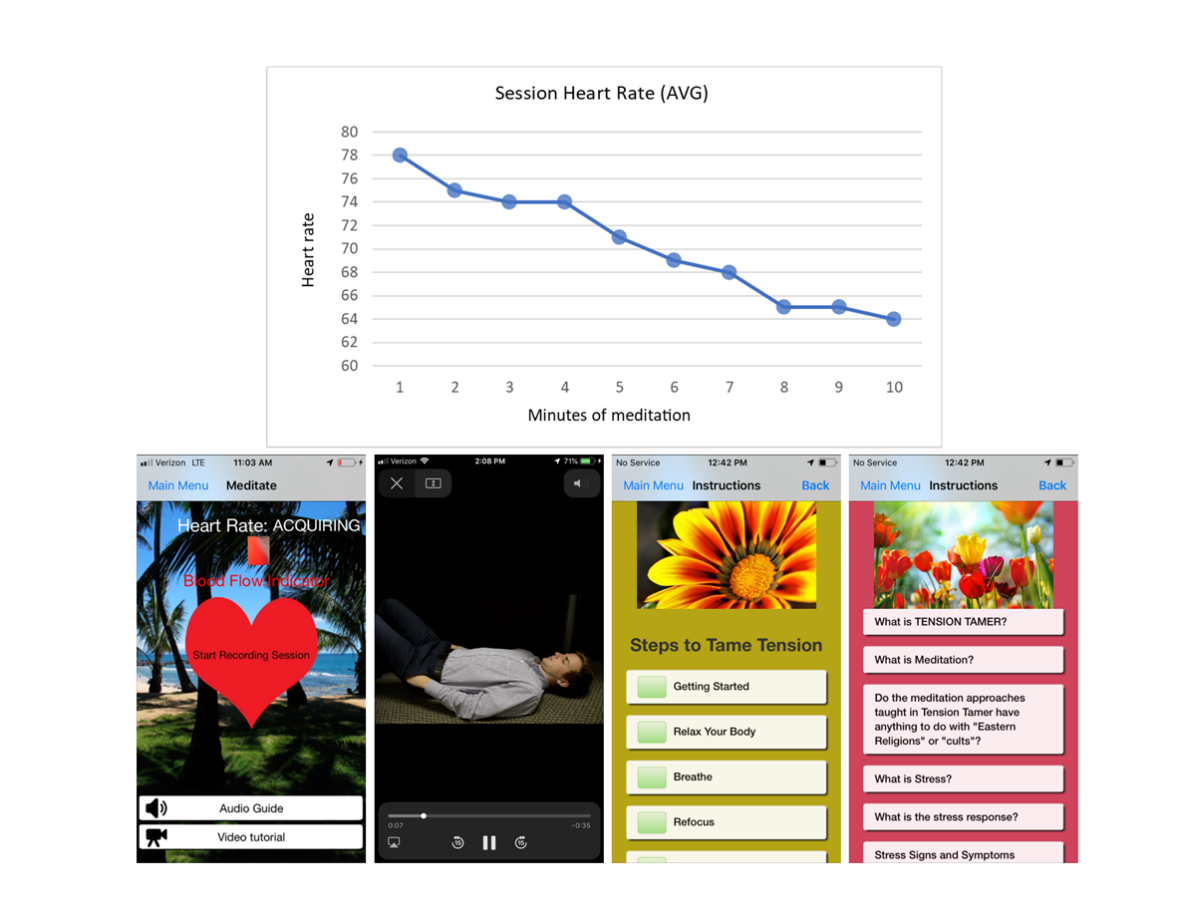
The graphical heart rate feedback after each Tension Tamer session to illustrate average heart rate changes across each minute using the smartphone’s camera (top). Screenshots of the home screen (bottom), breathing meditation instructions, information regarding how Tension Tamer works, and a frequently asked questions page.

Tension Tamer captures real-time heart rate (HR) with a proprietary software involving reflective photoplethysmography by using the smartphone video camera and light-emitting diode (LED) flashlight [45]. The video camera and software can detect HR pulses through the finger’s capillary beds and output the digital reading on the interface and store the session HR data. This feature has been previously validated against electrocardiogram-derived HR (Pearson r≥.99, standard error of the estimate≤2.09 bpm) [45]. Graphical HR feedback is provided after each Tension Tamer session to illustrate the average HR changes across each minute (Figure 1).

Users have the option to turn off (or reactivate) the audio BAM instructions, set session midway and ending alerts (chime or gong), and select different background themes for screenshots (eg, nature, rock garden, beach scenes; Figure 1). The Tension Tamer app sends duration and time stamp–encrypted HR data to our institution’s Health Insurance Portability and Accountability Act–compliant server for storage in a relational database management system via a securely authenticated web interface. The time-stamped HR data provide the duration of session data, which is used to facilitate the measurement of session adherence.

The Tension Tamer app was developed and tailored for prehypertensive adults using a patient-centered iterative design approach guided by behavioral change theories. These included the self-determination theory [46] and the social cognitive theory [47] to guide the development of the behavioral content and the *what*, *how*, and *why* of Tamer Tension engagement and maintenance by fostering self-efficacy and intrinsic motivation to sustain engagement in the meditation sessions over time [48]. The People, Activity, Context, and Technology (PACT) model guided the presentation and engagement process of using the app. The PACT model posits that users must feel at ease with and perceive the technology as useful in reaching a desired goal [49]. Feedback based on the levels of adherence in the Tension Tamer program was used to provide social reinforcement and motivation via SMS messages to increase self-efficacy and autonomous motivation to increase adoption and sustain engagement of Tension Tamer for the management of stress and BP. As noted earlier, the primary outcomes of changes in BP from the 6-month dose-response trial involving prehypertensive adults have been reported [23].

Sleep data were collected through self-reported surveys and 1-week continuous actigraphy acquisition at each evaluation time point (baseline and months 1, 3, and 6). All research procedures were approved by the institutional review board before conducting the study (IRB #Pro00020894).

### Procedures

Complete details of the study protocol have been reported elsewhere [23]. After informed consent was obtained, and 3 separate confirmatory BP evaluations were done to verify the prehypertensive status, baseline measures of demographics (ie, ethnicity, education, income, height, weight, and physical activity level) were taken and a set of questionnaires including the Pittsburgh Sleep Quality Index (PSQI) were administered [50]. BMI was calculated as weight in kilograms divided by height in meters squared. Participants also wore an ActiGraph monitor for 7 consecutive 24-hour periods. Participants were subsequently randomized into 1 of 3 Tension Tamer dosage conditions of 5, 10, or 15 min, each to be performed twice daily.

The Tension Tamer app was installed on either the iOS (iPhone operating system) or the Android operating system on each participant’s personal smartphone. If the subject did not have a smartphone, an Apple iPhone 4 or 5 or Samsung Galaxy 4 or 5 was provided at no cost for the duration of the study. A staff member trained in the use of the Tension Tamer app was present to provide guidance as needed during the introductory tutorial. The Tension Tamer app tutorial included Tension Tamer videos explaining BAM, demonstrating diaphragmatic breathing, and explaining how to operate the app’s features, including obtaining video camera HR capture using the fingertip. An audio recording of a British female voice guided them through their first BAM session. Participants were instructed to place the tip of a finger over the camera’s lens and LED light. The app would vibrate if the finger were misaligned and prompt for realignment. Participants were directed in the audio guide to sit comfortably, close their eyes, and attend to the movement of their diaphragm while taking deep, slow, *belly* breaths. At the start of each session, a chime indicated the start with an on-screen timer. Additional chimes notified the user of the midpoint and the end of the session. Numeric feedback was immediately displayed on a 4-beat rolling average basis throughout the session. A graph was displayed immediately following the session, showing the average HR response during each minute of the session (shown in Figure 1). The training session ended with the participant demonstrating the ability to initiate another Tension Tamer session, including HR acquisition, before returning home. In subsequent BAM sessions, the participants could toggle the guided audio training on or off as desired.

An ActiGraph monitor was then fitted on their wrist, and participants were given instructions to wear the device for 7 consecutive days and to complete a daily sleep log. The same procedure was followed at each subsequent laboratory evaluation at 1, 3, and 6 months. Participants were compensated US $145 for their time after completion of each evaluation.

### Measures

#### Actigraphy

Actigraphy was captured using the ActiSleep monitor (ActiGraph models GT3XP-BTLE and ASP-BTLE; ActiGraph Corp). The ActiSleep monitor is a research-grade accelerometer that provides objective estimates of physical movement and includes multiple sleep parameters that have been validated against polysomnography [51-54]. The wrist monitor was secured on the nondominant hand using the manufacturer’s Velcro wrist strap. Participants were told to wear the device at all times except during activities that would get the device wet (eg, showering, bathing, swimming). Epochs were set to 10-second intervals. Participants were given sleep logs for recording each night’s sleep time and wake time and to note any disturbances (eg, awakened by noise, having bad dreams, using the bathroom). Data were downloaded and processed using ActiLife 6 software (version 6.13.3, ActiGraph LLC), which converted the data to 60-second epochs. Initially, the software’s automated sleep detection algorithm was used to identify the sleep periods. Comparisons were performed against the sleep log data by a doctoral-level analyst, and adjustments were made when the sleep and wake times differed by more than 15 min. Sleep and wake changes were identified using visual examination of the graphical output, self-reported notes on sleep logs, nonwear periods, lux readouts from the light sensor, and patterns across all days. The Cole-Kripke algorithm [55] was used to categorize the sleep and wake status during each sleep period. The following variables were averaged at each time point and retained for analysis: sleep time per night (in min), sleep efficiency (ie, the percentage of time in bed that was spent asleep), and sleep fragmentation index (ie, an ActiSleep-derived index of restlessness during the entire sleep period by measurement of the amount of movement while in bed; lower values indicate better sleep). At least 4 days of valid data, including 1 weekend night, were required at each evaluation to be included in the analyses.

#### PSQI

PSQI, administered at each evaluation, is a well-validated self-report questionnaire of sleep quality over the previous month [50,56-59]. The 19 items on PSQI use both open-ended (eg, regular bedtime and awakening times) and 5-point Likert scale formats. Scoring algorithms provide 7 components (subjective sleep quality, sleep latency, sleep duration, sleep efficiency, sleep disturbances, sleep medication, and daytime dysfunction). The total sleep quality score (0-21) is derived from the 7 components (each scored 0-3). Higher scores on each component are indicative of poorer sleep quality. In addition to the 7 components, Cole et al [53] identified 3 latent factors based on a combination of PSQI components: sleep efficiency (sleep duration and sleep efficiency), perceived sleep quality (subjective sleep quality, sleep latency, and sleep medication), and daily disturbances (sleep disturbances and daytime dysfunction). A recent meta-analysis of 37 PSQI psychometric studies has revealed good internal consistency (Cronbach alpha=.70-.83), construct validity (ie, discriminated between groups with a disorder associated with poor sleep, such as depression and PTSD, and healthy individuals), convergent validity (eg, moderate positive correlations with anxiety and depression), and divergent validity (eg, weak associations with unrelated constructs such as vomiting and anger) [58]. The 3-factor structure has been replicated across a variety of populations involving men and women with various chronic illnesses or conditions (eg, depression, pregnant women, kidney transplant recipients, PTSD, perimenopause, early postmenopausal women) as well as those without any known diseases. Participants have represented multiple ethnic groups (eg, AAs, NHWs, Hispanic Americans) [59-62]. We used the 3 factors (sleep efficiency, sleep quality, and daily disturbances) along with the global score in the statistical analyses.

#### Physical Activity

In addition to the objective physical activity data provided by the ActiSleep monitor, a single-item self-report question was completed at each evaluation for comparison. The question, derived from the Sweat Index [63], was “How many days per week are you active enough where you begin to sweat?” Response options ranged from 0 to 7 days. Comparisons between baseline and follow-up were used to adjust modeling if activity levels increased or decreased significantly.

#### Adherence

Adherence was measured using the encrypted time and date stamped HR data obtained via the Tension Tamer app’s built-in photoplethysmogram that were automatically relayed to our institution’s server infrastructure. These data provided objective measures of adherence (ie, 1.0=2 sessions at full dose over a 24-hour period separated by >5 min; 0.5=completion of 1 session during the 24-hour period). Adherence was reported as a monthly percentage calculated as the average of daily scores (0, 0.5, and 1.0) across the number of days in the month.

### Statistical Analyses

Demographic variables were analyzed using the analysis of variance for continuous data and chi-square tests for categorical data. For all analyses, statistical significance was set at *P*=.05. Analyses of longitudinal dose intervention effects from baseline to final 6-month evaluation for self-reported (PSQI) and actigraphy sleep indices were performed on the intention-to-treat (ITT) sample comprising all participants who completed at least four Tension Tamer sessions following completion of the baseline evaluation (n=57). A generalized linear mixed (GLM) model approach was used to account for the correlation of repeated measurements within patients [64,65]. The GLM models used the available measures at all time points (baseline and months 1, 3, and 6) and summarized the difference between groups at the final measurement (ie, month 6). A 2-level analysis strategy was utilized. Our primary analyses included the simple mixed-effects model containing the intervention dose group, the primary variable of interest, along with time (study visit), a dose-by-study visit interaction term as the fixed effect and adjusted for the baseline measure of the dependent variable with an unstructured covariance structure. The model was further adjusted for adherence.

The second level of GLM modeling included ethnicity as a fixed effect in addition to the previously included effects because of the statistically significant differences in most actigraphy-derived sleep indices observed at baseline. We estimated changes in sleep for each subject over the trial (preintervention and 1, 3, and 6 months) and the within-subject longitudinal trajectories (eg, slopes) and summarized the mean longitudinal trajectory within each dose group.

All sleep outcome variables were examined for normality using the Shapiro-Wilk test. With 18 subjects randomized to each intervention group, we had 80% power to detect at least a 0.96 SD unit effect size among the 3 groups (level of significance [alpha]=.05, 2-tailed) for sleep outcomes. SAS Statistical Software version 9.4 (SAS Institute Inc) and SPSS Statistics version 25.0 (IBM Corp) were used to perform the analyses.

## Results

### Overview

**Figure 2:**
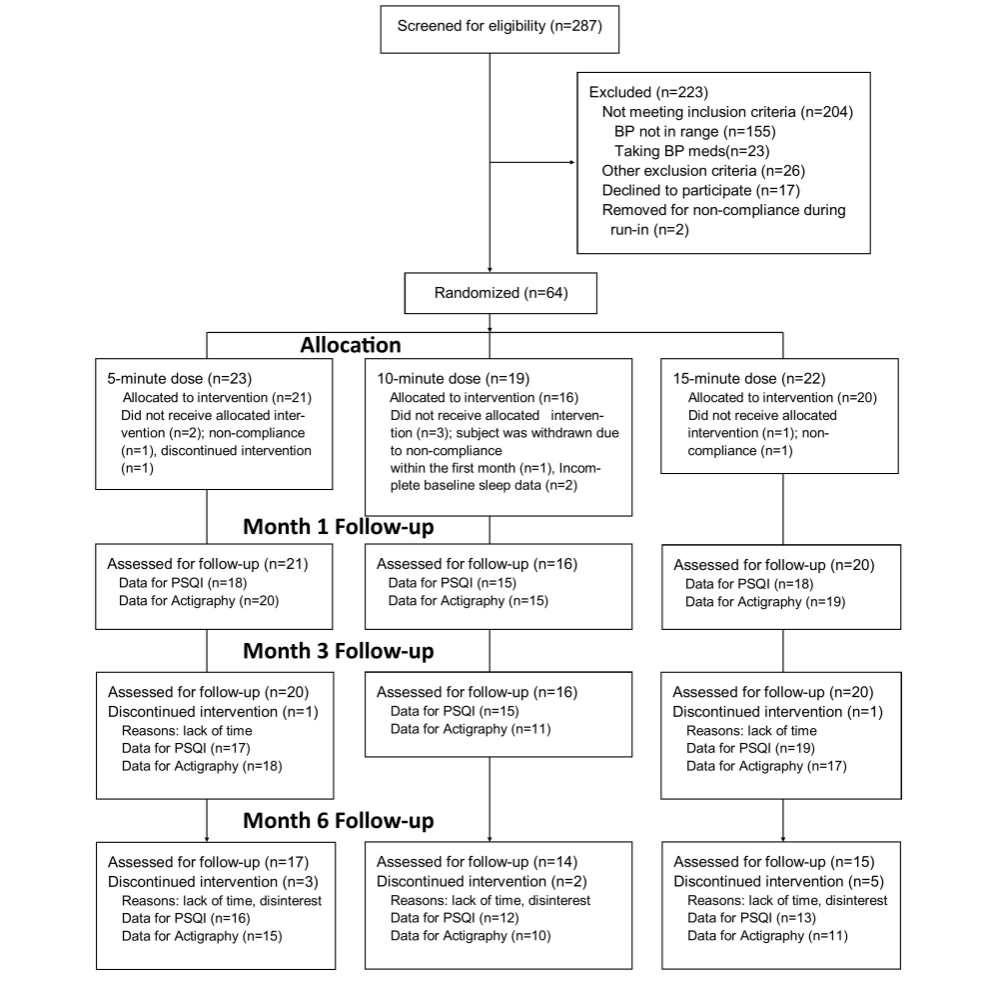
Recruitment and assessment flow diagram. PSQI: Pittsburgh Sleep Quality Index.

The study was approved in July 2014 (IRB#Pro00020894) and was performed between August, 2014 and October, 2016. A total of 287 potential candidates were screened (Figure 2). Overall, 204 patients were found to be ineligible. The primary reasons for exclusion included not meeting prehypertension criteria on all 3 screening visits (155/204, 76.0%), taking prescription medications that influenced BP (23/204, 11.2%), and failure to meet various other inclusion criteria (26/204, 12.7%), Of the 83 eligible participants, 64 (77%) consented to participate in the clinical trial. A total of 2 participants subsequently failed to complete the baseline evaluation monitoring in the natural environment. Furthermore, 5 others failed to complete at least 4 sessions during the first several weeks and dropped out (n=4) or were removed from the trial (n=3). These collective 7 individuals were evenly distributed across dosage conditions.

### Baseline Characteristics

Overall, 47% (27/57) of the participants were female and 49% (28/57) were African American. As illustrated in Table 1, there were no significant baseline differences among the dosage groups on any descriptive characteristics (eg, education levels, income, BMI, physical activity, or stress; all values of *P*=.07 or greater). In addition, baseline sleep characteristics were not significantly different among dosage groups for any self-reported or objective variable (all *P* values were greater than .20).

AAs had higher BMI, reported less sweat-induced physical activity, and had lower education levels compared with NHWs (*P*=.04 to *P*=.001). Importantly, ethnicity differences were observed in 6 of the 7 baseline sleep quality indices (Table 2).

**Table 1:** Baseline demographic and sleep characteristics by dosage condition.

Characteristics		Overall (n=57)	Dose			*P* value^a^	
			5 min (n=21)	10 min (n=16)	15 min (n=20)		
Age (years), mean (SD)		34.98 (12.79)	35.8 (11.10)	37.8 (13.9)	31.85 (13.48)		.36
Gender (female), n (%)		27 (47)	11 (52)	8 (50)	8 (40)		.71
Ethnicity (African American), n (%)		28 (49)	12 (67)	8 (50)	8 (40)		.55
BMI (kg/m^2^), mean (SD)		27.63 (5.7)	28.5 (7.00)	26.4 (4.59)	27.7 (4.9)		.57
Physical activity (days/week), mean (SD)		3.32 (216)	3.1 (2.08)	3.1 (2.13)	3.7 (2.1)		.65
**Annual income (US$), n (%)**						.28	
	<15,000	20 (35)	6 (29)	4 (25)	10 (50)		
	15,000-29,999	20 (35)	7 (33)	7 (44)	6 (30)		
	30,000-49,999	10 (18)	4 (19)	4 (25)	2 (10)		
	50,000-74,999	2 (4)	0 (0)	0 (0)	2 (10)		
	>75,000	1 (2)	1 (5)	0 (0)	0 (0)		
	Not reported	4 (7)	3 (14)	1 (6)	0 (0)		
**Education, n (%)**						.92	
	High school	5 (9)	2 (10)	2 (13)	5 (1)		
	Trade school	10 (18)	5 (24)	2 (13)	3 (15)		
	College educated	32 (56)	10 (48)	9 (56)	13 (65)		
	Not reported	10 (18)	4 (19)	3 (19)	3 (15)		
**Actigraphy indices, mean (SD)**							
	Sleep efficiency (%)	86.97 (6.62)	87.74 (5.45)	88.06 (6.40)	85.32 (7.82)		.40
	Sleep duration (min/night)	380.24 (61.96)	379.88 (65.44)	373.17 (76.08)	386.20 (46.92)		.84
	Sleep fragmentation (units)	26.76 (8.24)	25.67 (8.56)	25.06 (6.86)	29.26 (8.69)		.26
	Valid days (days)	6.80 (0.49)	6.95 (0.22)	6.73 (0.59)	6.68 (0.58)		.20
**Pittsburgh Sleep Quality Index domains, mean (SD)**							
	Sleep quality	2.3 (1.4)	1.9 (1.4)	2.3 (1.5)	2.6 (1.4)		.23
	Sleep efficiency	1.3 (.96)	1.2 (1.1)	1.5 (1.0)	1.2 (.8)		.50
	Daily disturbances	1.9 (1.0)	1.9 (1.1)	2.0 (1.2)	2.1 (.9)		.76
	Global score	5.6 (2.6)	5.0 (2.8)	5.8 (2.7)	5.9 (2.4)		.44

^a^*P* values were determined by using the analysis of variance and the chi-square test (n=57).

**Table 2:** Baseline demographic and sleep characteristics by ethnicity.

Characteristics		Non-Hispanic White (n=29)	African American (n=28)	*P* value^a^
Age (years), mean (SD)		32.07 (10.84)	38.00 (14.10)	.08
Gender (female), n (%)		11 (38)	16 (57)	.15
BMI (kg/m^2^), mean (SD)		25.47 (4.12)	29.87 (6.20)	.003
Physical activity (days/week), mean (SD)		3.86 (2.21)	2.74 (1.75)	.04
**Annual income (US$), n (%)**				.31
	<15,000	45 (13)	7 (25)	
	15,000-29,999	9 (31)	11 (39)	
	30,000-49,999	5 (17)	5 (18)	
	50,000-74,999	0 (0)	2 (7)	
	>75,000	1 (3)	0 (0)	
	Not reported	1 (3)	3 (11)	
**Education, n (%)**				.001
	High school	0 (0)	5 (8)	
	Trade school	1 (3)	9 (32)	
	College educated	21 (72)	11 (39)	
	Not reported	7 (24)	3 (11)	
**Actigraphy indices, mean (SD)**				
	Sleep efficiency (%)	89.36 (4.71)	84.21 (7.48)	.003
	Sleep duration (min/night)	413.25 (42.58)	341.00 (59.33)	.001
	Sleep fragmentation (units)	24.34 (5.39)	29.57 (10.03)	.02
**Pittsburgh Sleep Quality Index** **domains, mean (SD)**				
	Sleep quality	1.9 (1.4)	2.7 (1.3)	.03
	Sleep efficiency	0.8 (0.7)	1.8 (0.9)	<.001
	Daily disturbances	2.0 (1.1)	2.0 (1.0)	.89
	Global score	4.7 (2.5)	6.5 (2.5)	.008

^a^*P* values were calculated using the analysis of variance and chi-square tests (n=57).

On the basis of the PSQI, AAs self-reported significantly poorer sleep efficiency (ie, shorter average sleep duration, higher percentage of time awake while trying to sleep) and poorer sleep quality (ie, using sleep medications, longer latency, poorer overall sleep quality); *P*<.001 and .03, respectively. As expected, their PSQI global sleep quality scores were higher (*P*=.008), with 50% (14/28) falling above the clinical cutoff level for sleep disturbance (PSQI global score >5) [50,58,61] compared with 13% (4/29) among NHWs. Importantly, all 3 actigraphy-derived indices supported the self-reported findings, with AAs exhibiting poorer sleep efficiency, greater fragmentation, and shorter average sleep duration than NHWs (*P*=.02 to *P*=.001).

### Impact of Tension Tamer dosage on Sleep

Longitudinal GLM models evaluating the impact of Tension Tamer dosage conditions on sleep variables at 1, 3, and 6 months, controlling for baseline levels and adherence, did not reveal any statistically significant dose×time interaction terms in any of the PSQI-based sleep outcomes (all *P*=.08 or greater). Among the ActiGraph-derived variables, a significant dose effect was observed only for sleep fragmentation (*P*=.03). Collapsed across the 3 evaluation time points, the 5-min dose condition exhibited the lowest sleep fragmentation (23.4, SD 1.2) compared with both the 10-min (28.3, SD 1.5; *P*<.001) and 15-min (28.7, SD 1.3; *P=*.006) dose conditions.

### Impact of Tension Tamer Dosage on Sleep Measures Within Ethnic Groups

GLM modeling was conducted with ethnicity as a covariate in the model (Table 3). Ethnicity was found to have a significant impact on the models’ dosage results on 2 of the 3 actigraphic measures (fragmentation and efficiency; for both, *P*=.01) and on the PSQI sleep efficiency factor (*P=*.03).

**Table 3:** Effect of the dosage of Tension Tamer app on sleep measures within ethnic groups (adjusted means with SE).

Characteristics				Dose, 5 min		Dose, 10 min		Dose,15 min		Diff 5-10		*P* value		Diff 5-15		*P* value		Diff 10-15		*P* value
**Sleep outcomes for non-Hispanic Whites^a^**																				
	**Actigraphy indices, mean (SE)**																			
		Sleep efficiency (%)	91.4 (1.2)		87.2 (1.4)		89.7 (1.3)		4.2 (1.8)		.03		1.7 (1.8)		.35		−2.5 (1.9)		.19	
		Sleep fragment	21.9 (2.3)		26.1 (2.7)		25.5 (2.6)		−4.2 (1.8)		.25		−3.6 (3.5)		.32		0.6 (3.7)		.87	
		Sleep duration (min/night)	419.9 (12.8)		403.5 (14.7)		403.1 (14.0)		16.4 (19.5)		.41		16.8 (19.3)		.39		0.40 (19.8)		.98	
	**PSQI^b^, mean (SE)**																			
		Sleep quality	0.7 (0.3)		1.5 (0.3)		2.2 (0.4)		−0.7 (0.5)		.02		−1.5 (0.5)		.006		−0.8 (0.5)		.13	
		Sleep efficiency	0.3 (0.2)		0.9 (0.2)		0.9 (0.2)		−0.6 (0.3)		.11		−0.7 (0.3)		.02		−0.1 (0.2)		.83	
		Daily disturbances	1.4 (02)		1.6 (0.3)		2.1 (0.3)		−0.2 (0.4)		.57		−0.7 (0.4)		.09		−0.5 (0.4)		.23	
		Global score	2.5 (0.4)		4.0 (0.4)		5.2 (0.4)		−1.4 (0.6)		.03		−2.7 (0.6)		<.001		−1.3 (0.6)		.04	
**Sleep outcomes for African Americans**																				
	**Actigraphy indices, mean (SE)**																			
		Sleep efficiency (%)	85.9 (2.0)		82.9 (2.7)		83.0 (2.6)		3.0 (3.3)		.38		2.9 (3.2)		.38		−0.1 (3.8)		.99	
		Sleep fragment	24.5 (1.7)		30.9 (2.2)		37.4 (2.3)		−6.4 (2.7)		.03		−12.0 (3.1)		<.001		−6.7 (3.4)		.07	
		Sleep duration (min/night)	334.2 (9.9)		374.6 (12.6)		294.0 (13.3)		−40.5 (15.9)		.02		40.1 (17.0)		.03		80.6 (19.1)		<.001	
	**PSQI, mean (SE)**																			
		Sleep quality	2.2 (0.6)		2.2 (0.6)		1.7 (0.8)		−0.0 (0.9)		.99		0.4 (0.9)		.64		0.4 (0.9)		.63	
		Sleep efficiency	2.0 (0.3)		1.6 (0.3)		2.0 (0.3)		0.4 (0.4)		.35		0.04 (0.5)		.93		−0.4 (0.5)		.44	
		Daily disturbances	1.6 (0.4)		2.2 (0.4)		1.1 (0.5)		−0.6 (0.6)		.29		0.5 (0.6)		.64		1.1 (0.6)		.08	
		Global score	6.0 (1.0)		6.0 (1.0)		4.9 (1.2)		0.0 (1.5)		.99		1.1 (1.6)		.50		1.1 (1.5)		.50	

^a^Differences determined by longitudinal generalized linear mixed modeling adjusted for visit, dose-by-visit interaction, adherence, and baseline outcome level (follow-up time points include 1, 3, and 6 months).

^b^PSQI: Pittsburgh Sleep Quality Index.

As a result of the above findings, an ethnicity×dose interaction term was added to the model to determine whether ethnicity modulated the effects of dosage level on sleep outcomes. Significant interaction effects were observed for 2 of the 3 actigraphic measures (efficiency *P*=.05; duration *P*=.03). The sleep measure was the dependent variable, whereas dosage was the primary independent variable adjusted for visit, dose-by-visit interaction term, baseline level of particular outcome measure, and adherence.

The NHWs exhibited a consistent pattern across both self-reported and ActiGraph-derived measures. The 5-min dose resulted in the greatest improvements in sleep measures compared with the other 2 dosage conditions. On the basis of the PSQI data, participants in the 5-min condition reported significantly better values for the sleep efficiency factor compared with both the 10-min and 15-min conditions (for both, *P*=.02). Similarly, they also reported better scores for the sleep quality factor compared with the 10-min and 15-min dose conditions, with the difference for the 15-min condition being statistically significant (*P*=.006). Finally, the 5-min condition reported significantly better global sleep quality compared with both the 10-min and 15-min conditions (*P*=.03 and <.001, respectively).

The ActiGraph findings supported these patterns among NHWs, with the 5-min group showing higher sleep efficiency compared with the 10-min (*P*=.03) and 15-min groups; however, the latter difference did not reach statistical significance. Similarly, the 5-min condition exhibited lower fragmentation and longer sleep duration compared with the other 2 conditions, but the differences were not statistically significant.

Among AAs, the actigraphy findings indicated that the 5-min condition exhibited significantly lower sleep fragmentation than both the 10-min and 15-min conditions (*P*=.03 and *P*<.001, respectively). The 10-min condition showed the highest sleep duration, which was significantly longer than both the 5-min and 15-min conditions (*P*=.02 and *P*<.001, respectively). The 5-min condition also exhibited significantly longer sleep duration compared with the 15-min condition (*P*=.03).

With regard to the self-reported results among AAs, none of the PSQI factor scores were significantly and differentially impacted by the Tension Tamer dosage, except for PSQI daily disturbances. The 15-min group showed a trend of lower daily disturbances compared with the 10-min group (*P*=.08). Furthermore, the overall pattern of PSQI factor scores did not emulate the actigraphy findings. For example, the 15-min dose condition, which had the shortest actigraphy-derived sleep duration and greatest fragmentation during sleep, had the best sleep quality factor, global sleep quality scores, and daily disturbance factor score.

### Effect of Differences in Tension Tamer Dosages on Sleep Measures Between Ethnic Groups

Further modeling was carried out to examine potential ethnicity differences in response to the 3 dosage levels given in Table 4. Adjusted means and SE by ethnicity within each dosage level at month 6 were obtained from longitudinal GLM modeling. The model included sleep measure as the dependent variable and dose as the primary independent variable of interest adjusted for visit, ethnicity, dose-by-visit and dose-by-visit-by-ethnicity interaction terms, baseline value of sleep measure, and adherence.

Per the PSQI outcomes, NHWs reported a consistent pattern of greater improvement than AAs for each PSQI factor (sleep quality, sleep efficiency, and daily disturbances) and global sleep quality for the 5-min and 10-min dose conditions. However, the only statistically significant differences observed were for the 5-min dose condition, in which NHWs reported greater sleep efficiency (*P*=.002) and better global sleep quality (*P*=.05) than AAs. The 15-min dose condition revealed a different pattern in which AAs reported better scores in sleep quality and daily disturbance factors and lower global sleep quality scores than NHWs; however, none of these differences reached statistical significance (all values of *P*=.18 or greater).

The ActiGraph results revealed a pattern in which NHWs exhibited comparable or better sleep efficiency and fragmentation than AAs across all 3 doses. They also exhibited longer sleep durations at the 5-min and 15-min dose conditions. However, statistically significant differences were observed in the 15-min dose condition only. NHWs exhibited better efficiency (*P*=.02), lower fragmentation (*P*=.02), and longer sleep duration (*P*=.002) than AAs.

**Table 4:** Effect of ethnicity differences and the dosage of Tension Tamer app on sleep measures at 6-month follow-upa. Values represent the adjusted means and SE.

Characteristics			Non-Hispanic Whites		African Americans		*P* value	
**Sleep outcomes for 5-min dose^a^**								
	**Actigraphy, mean (SE)**							
		Sleep efficiency (%)		91.4 (1.2)		85.9 (2.0)		.18
		Sleep fragment		21.9 (2.3)		24.5 (1.7)		.93
		Sleep duration (min/night)		419.9 (12.8)		334.2 (9.9)		.13
	**PSQI** ^b^ **, mean (SE)**							
		Sleep quality		0.7 (0.3)		2.2 (0.6)		.14
		Sleep efficiency		0.3 (0.2)		2.0 (0.3)		<.001
		Daily disturbances		1.4 (02)		1.6 (0.4)		.56
		Global score		2.5 (0.4)		6.0 (1.0)		.05
**Sleep outcomes for 10-min dose**								
	**Actigraphy, mean (SE)**							
		Sleep efficiency (%)		87.2 (1.4)		82.9 (2.7)		.66
		Sleep fragment		26.1 (2.7)		30.9 (2.2)		.32
		Sleep duration (min/night)		403.50 (14.7)		374.6 (12.6)		.28
	**PSQI, mean (SE)**							
		Sleep quality		1.5 (0.3)		2.2 (0.6)		.57
		Sleep efficiency		0.9 (0.2)		1.6 (0.3)		.60
		Daily disturbances		1.6 (0.3)		2.2 (0.4)		.13
		Global score		4.0 (0.4)		6.0 (1.0)		.32
**Sleep outcomes for 15-min dose**								
	**Actigraphy, mean (SE)**							
		Sleep efficiency (%)		89.7 (1.3)		83.0 (2.6)		.02
		Sleep fragment		25.5 (2.6)		37.4 (2.3)		.02
		Sleep duration (min/night)		403.1 (14.0)		294.0 (13.3)		.002
	**PSQI, mean (SE)**							
		Sleep quality		2.2 (0.4)		1.7 (0.8)		.48
		Sleep efficiency		0.9 (0.2)		2.0 (0.3)		.12
		Daily disturbances		2.1 (0.3)		1.1 (0.5)		.18
		Global score		5.2 (0.4)		4.9 (1.2)		.54

^a^Differences determined by longitudinal generalized linear mixed modeling adjusted for visit, dose-by-visit interaction, adherence, and baseline outcome level.

^b^PSQI: Pittsburgh Sleep Quality Index.

## Discussion

### Principal Findings

This is the first study to use a smartphone app to examine the effect of varying doses of BAM on self-reported and objective indices of sleep. Baseline measurements corroborated the literature with regard to adult ethnicity differences in sleep quality [27-31,35,57-59,66]. Similar to most research that has utilized self-reported methodology, we used the well-validated PSQI survey [50,56] and a 3-factor model first reported by Cole et al [57] and replicated it across multiple patient populations and ethnic groups, including NHWs and AAs [59-61]. AAs, compared with NHWs, reported statistically significant poorer scores for sleep efficiency (ie, shorter average sleep duration, higher percentage time awake while trying to sleep) and sleep quality (ie, using sleep medications, longer latency, poorer general sleep quality) factors. On the basis of the clinical cutoff point for poor sleep quality (ie, PSQI global score of >5) [50,58,61], AAs had a significantly higher prevalence of poor sleep quality compared with NHWs.

The 24-hour monitoring with the ActiGraph wrist monitor for 7 consecutive days at each evaluation point provided objective indices of sleep. Importantly, these data corroborated the baseline self-reported findings in that AAs exhibited shorter sleep duration, more fragmented sleep, and lower sleep efficiency.

Longitudinal modeling analyses indicated that the BAM program, delivered and monitored using the Tension Tamer smartphone app, had a beneficial impact on sleep, which was modulated by ethnicity. On the basis of actigraphy monitoring, among AAs, the 5-min dose resulted in significantly lower fragmentation compared with both the 10-min and 15-min dose conditions. The 5-min dose group also had significantly longer sleep duration than the 15-min dose group; however, the 10-min dose resulted in the longest sleep duration compared with both the 5-min and 15-min doses. Previously, we compared the changes in SBP among dosage conditions and observed that the 15-min condition had significantly lower adherence rates compared with the other 2 conditions [23]. By 6 months, the ITT analysis using 75% adherence criterion found that the 5-min condition had higher percentages (57%) meeting this criterion compared with 37% for the 10-min condition and 14% for the 15-min condition.

Among the AAs, no significant differential impact by dosage condition was observed on self-reported sleep. Furthermore, the general pattern of PSQI-derived sleep dimensions did not corroborate the actigraphy findings. This discrepancy is akin to the findings that AAs exhibit lower correlations between self-reported sleep duration and both actigraphy- and polysomnography-derived sleep durations (r range 0.15 to 0.29) compared with NHWs (r range 0.45 to 0.56) [33,67-69].

On the other hand, among the NHWs, a consistent pattern across both self-reported and ActiGraph-derived indices indicated that the 5-min dose resulted in the greatest improvements in sleep measures. On the basis of the PSQI, the 5-min condition reported significantly greater improvements in sleep efficiency compared with both the 10-min and 15-min conditions. Similarly, the 5-min condition reported better sleep quality compared with the other 2 conditions, with the difference for the 15-min condition being statistically significant. Finally, the 5-min dose condition reported significantly better PSQI global scores (summation of the original 7 sleep components) compared with both 10-min and 15-min dose conditions. The pattern of actigraphy findings supported the self-reported findings. The 5-min condition exhibited higher sleep efficiency scores compared with both 10-min and 15-min conditions, with the 10-min comparison being statistically significant. Similarly, the 5-min condition had lower fragmentation compared with the other 2 dosage conditions; however, the differences did not reach statistical significance.

Evaluations of potential ethnicity differences in response to the 3 Tension Tamer doses revealed a consistent pattern of significantly better responsivity among the NHWs. ActiGraph-derived measures indicated significantly greater improvements among NHWs in sleep efficiency, reduced fragmentation, and longer sleep duration compared with AAs at each dosage condition. The PSQI findings provided support to the actigraphy findings, with NHWs reporting a pattern of higher quality, efficiency, and better PSQI global sleep scores for both 5-min and 10-min dose conditions compared with AAs. Compared with NHWs, AAs in the 15-min dose condition reported better sleep quality and PSQI daily disturbance scores, with a trend toward better global sleep scores. A higher use of Tension Tamer in the 5-min group across the trial may have contributed to the generally greater beneficial influence on sleep quality. Anecdotally, the 5-min participants appeared to more often note at posttrial key informant interviews using Tension Tamer immediately before going to bed, which may have played a role as well.

As noted earlier, the findings have been mixed with regard to the impact of meditation on sleep [9,11,13,17,24]. The overall results of this study provide increasing support for the role of smartphone-enabled meditation and the benefits of multimethod-based sleep outcomes among individuals not originally recruited for sleep disturbance studies [70-72]. As pointed out in a review of 123 mind-body based interventions by Neuendorf et al [11], the use of meditation may be advantageous to improve sleep among individuals who do not have clinical levels of sleep disturbance, but the observed effect sizes are likely to be less. The mixed sleep results observed in earlier studies may be due to poor scientific rigor, lack of measurement of adherence, or dosage of mindfulness-based practices, especially in studies conducted as home-based programs [13]. These shortcomings are addressed in this report where dosage and adherence measures bolster the quality of our findings.

Use of cellular technology provides the possibility of disseminating efficacious mind-body intervention programs such as BAM to larger audiences, circumventing barriers such as travel, childcare, and other expense-related issues. In addition, the penetration of smartphone-assisted technology into lower income, rural, and urban areas enables these programs to be widely disseminated and available. This is increasingly important as 81% of adults own a smartphone as of June 2019 [73]. Our exit surveys suggest that Tension Tamer sessions conducted before bedtime may aid individuals in going to sleep faster. Thus, engagement with Tension Tamer as part of the sleep preparation ritual may be beneficial for future studies. Tension Tamer may potentially have a larger effect in improving sleep if used as a direct intervention or in combination with other sleep hygiene practices on those exhibiting sleep complaints. Further iterative, user-centered design is needed with NHWs and AAs with sleep disturbances to understand their levels of sleep hygiene literacy and identify potential needs for culturally attuned tailoring of a refined Tension Tamer app that would include sleep hygiene education, video testimonies by NHWs and AAs who had experienced sleep issues and benefited from Tension Tamer, and video segments by such individuals showing how to use a refined Tension Tamer. This aligns with recent recommendations by Johnson et al [68], who emphasized the need for culturally sensitive efforts to reduce racial and ethnic sleep disparities. The study emphatically reports the disparities in AAs among the sleep dimensions of duration, quality, and sleepiness. Specifically, sleep programs involving ethnic minorities will likely need to include sleep hygiene education to help eradicate false beliefs on managing issues such as long sleep latency (eg, to fall asleep, just watch TV in bed or drink alcohol).

### Limitations

A limitation of this study was that the Tension Tamer app was not designed to specifically target individuals with sleep issues but was rather for those with prehypertension. As a result, we did not include eligibility criteria related to sleep problems, insomnia, or collect sleep medication usage. Therefore, bias toward the null hypothesis may be an explanation for some of the findings. In future studies, inclusion of targeted intervention materials in addressing sleep-related barriers in prehypertensive patients may have a greater effect. Such tactics could include increasing basic sleep hygiene awareness or using the Tension Tamer app in combination with cognitive behavioral therapy [68]. Furthermore, 2 other limitations, the lack of a control arm and the relatively small sample size in each dosage arm, limit the generalizability of the findings. Comparisons between ethnic groups also found that the AA sample was older, had higher BMIs, and lower levels of physical activity, which could have contributed to poorer sleep quality results.

This study was also not designed to examine the daily timing of BAM sessions around bedtime routines. A properly powered trial using a refined Tension Tamer app, including BAM as part of bedtime preparation rituals, is needed to understand how the timing and dosage of BAM or other meditation techniques may provide the most beneficial effect on sleep outcomes. We also did not investigate any related mechanistic evaluations such as electromyography or other common sleep tests such as polysomnography [74], which could impart important information on how BAM affects sleep using causal pathways.

### Conclusions

To our knowledge, this is the only study that has examined the influence of varying doses of meditation delivered via a smartphone app on multimethod-derived indices of sleep (ie, self-report and actigraphy) in a multiethnic population not diagnosed with sleep disturbance issues. A high level of methodological rigor was incorporated, including random assignment to dosage condition, automated feedback of dosage adherence, and postsession HR graphs to provide reinforcement for engaging with the Tension Tamer app. Ethnicity was found to play a modulating role on the effects of Tension Tamer. Formative evaluation adapted from the development of Tension Tamer [75] can target ethnicity-related sleep issues utilizing a theory-guided, user-centered iterative design. This could aid in using the Tension Tamer app to facilitate sleep hygiene education, dispel myths, incorporate sleep ritual behaviors, and further address ethnic sleep disparities, as suggested by Johnson et al [68]. Future studies should further investigate the potential for ethnicity-induced differences in self-reported and objectively derived sleep measures using varying doses of BAM or other types of meditation or cognitive behavioral sleep programs, especially among those with verified clinical levels of sleep disturbance. Issues to address include levels of adherence to the regimen, potential influence of timing of daily engagement, and how it may differentially affect barriers to sleep. Finally, future studies will benefit from identifying the underlying causal mechanisms for BAM’s beneficial impact on sleep (eg, alterations in sleep stages, HR variability, hypothalamic-pituitary-adrenal axis, sympathetic nervous system activation, rumination, worry, intrusive thoughts) [76].

## Abbreviations

AA: African Americans
BAM: breathing awareness meditation
BP: blood pressure
GLM: generalized linear mixed
HIPAA: Health Insurance Portability and Accountability Act
HR: heart rate
ITT: intention-to-treat
LED: light-emitting diode
NHW: non-Hispanic Whites
NIH: National Institutes of Health
PACT: People, Activity, Context, and Technology
PSQI: Pittsburgh Sleep Quality Index
PTSD: post-traumatic stress disorder
RCT: randomized controlled trial
SBP: systolic blood pressure
TM: transcendental meditation
